# Total surgical time in laparoscopic supracervical hysterectomy with laparoscopic in-bag-morcellation compared to laparoscopic supracervical hysterectomy with uncontained morcellation

**DOI:** 10.52054/FVVO.14.1.006

**Published:** 2022-04-03

**Authors:** H Krentel, G Tchartchian, L.A. Torres de la Roche, R.L. De Wilde

**Affiliations:** Department of Gynecology, Obstetrics and Gynecological Oncology, Bethesda Hospital, Academic Teaching Hospital, Duisburg, Germany; MIC Clinic, Berlin, Germany; Clinic of Gynecology, Obstetrics and Gynecological Oncology, University Hospital for Gynecology, Pius-Hospital Oldenburg, Medical Campus University of Oldenburg, Germany

**Keywords:** Laparoscopic supracervical hysterectomy, electric power morcellation, in-bag-morcellation, cell dissemination, tissue retrieval

## Abstract

**Background:**

A possible solution to the problem of cell dissemination through laparoscopic uncontained morcellation during laparoscopic supracervical hysterectomy (LASH) is the use of laparoscopic in-bag morcellation. One criticism regarding the use of in-bag morcellation is the additional surgical time associated with this procedure.

**Objectives:**

In this retrospective study we compared the total surgical time in LASH with laparoscopic in-bag morcellation (107 cases from 2016-2018) and LASH with uncontained morcellation (47 cases from 2015-2017).

**Materials and Methods:**

All surgeries were performed in the same department of minimally invasive gynaecological surgery by a total of three experienced surgeons for the indication of bleeding disorder and / or dysmenorrhea.

**Main outcome measures:**

We measured and compared total surgical time, surgical outcome, blood loss and complications in LASH with in-bag morcellation and with uncontained morcellation.

**Results:**

Total surgical time in both procedures do not show a significant difference. Considering the learning curve in laparoscopic bag use, the total surgical time in LASH with laparoscopic in-bag morcellation is shorter than total surgical time in LASH with uncontained morcellation. Laparoscopic in-bag morcellation consumes time for bag use and handling, but saves time as it eliminates the need for meticulous sampling of lost tissue fragments and the complex lavage of the peritoneal cavity after morcellation. There is no difference between both groups in terms of blood loss, complications and surgical results.

**Conclusion/What is new?:**

We conclude that LASH with in-bag morcellation is not related to additional surgical time when compared to LASH with uncontained morcellation.

## Introduction

For benign pathologies such as symptomatic uterine myomatosis and adenomyosis causing bleeding disorders and dysmenorrhea, laparoscopic supracervical hysterectomy (LASH) with electric power morcellation is a popular minimally invasive technique for hysterectomy. Tchartchian et al (2018a) showed a significant reduction in pain (dysmenorrhea and dyspareunia) and bleeding severity as well as high satisfaction rates for patients undergoing LASH (Tchartchian et al, 2013). The procedure is associated with low major (0.3 %) and minor (approx. 1.0 %) complication rates (Krentel and De Wilde, 2016; Donnez et al., 2009; Bojahr et al., 2006). LASH is also a feasible surgical method for very large uteri (Tchartchian et al., 2018b). The retrieval of the uterine body after laparoscopic supracervical resection can be achieved through mini-laparotomy, posterior colpotomy, laparoscopic uncontained morcellation or laparoscopic contained morcellation. Mini-laparotomy and colpotomy have been associated with additional possible intraoperative and postoperative complications including infection and pain. In order to maintain the advantages of the minimally invasive approach in LASH, laparoscopic intraabdominal morcellation of the uterine tissue and the use of port incisions for tissue retrieval are required. Direct intraoperative complications during morcellation, such as vessel or bowel injury, are rare (Bojahr et al., 2015), but uncontained morcellation has been associated with intrabdominal cell dissemination. Depending on the pathology of the morcellated uterine tissue, this can cause parasitic peritoneal or retroperitoneal myomatosis (Takeda et al., 2007; Miyake et al., 2009), new onset peritoneal or retroperitoneal adenomyosis, or endometriosis (Hilger and Magrina, 2006; Sepilian and Della Badia, 2003); a delayed malignant transformation of initially benign uterine tissue fragments years following surgery (Kill et al., 2011; Heaps et al., 1990); or a dissemination of occult uterine malignancies, like uterine sarcoma or endometrial cancer combined with a possible upstaging of the disease and a worsening of the prognosis (Park et al., 2011; Perri et al., 2009).

In 2014, the Food and Drug Administration warned against the use of electric power morcellation in hysterectomy and myomectomy (McCarthy, 2014). Subsequently various authors retrospectively analysed thousands of cases of LASH and reported an overall occult malignancy rate of 0.13 % to 2.4 % (Bojahr et al., 2015; Brown et al., 2015; Lieng et al., 2015; Brölmann et al., 2015; Sizzi et al., 2018). The rate of parasitic myoma after laparoscopic morcellation was reported by Van der Meulen et al. (2015) to confer an overall risk of 0.12 – 0.95 %. The risk of new peritoneal endometriosis after LASH with intraabdominal morcellation was 1.4 % in a comparative study by Schuster et al. (2012). These risks can be minimised by the use of laparoscopic in-bag-morcellation with bag systems especially designed for this purpose (Cholkeri-Singh and Miller, 2015, Paul et al., 2015, Rimbach et al., 2015).

### Aim and Hypothesis

To compare the parameters of surgical time, blood loss, surgical outcome and major complications in LASH with laparoscopic in bag morcellation versus laparoscopic uncontained morcellation and to show that LASH with contained morcellation is safer than and at least as fast as LASH with uncontained morcellation.

## Materials and Methods

We performed a retrospective comparative analysis of laparoscopic supracervical hysterectomies with laparoscopic in-bag morcellation and laparoscopic uncontained morcellation in patients treated in a single institution by three different gynaecological surgeons from 2015 - 2018. We analysed blood loss, major complication rate and overall surgical time from the first incision to the last suture, taking into account uterine weight, histopathology and indication for surgery in both groups. 47 patients who underwent LASH without bag use and 107 patients who received LASH with in-bag morcellation were included. All surgeries have been carried out in the same department following a standardised procedure. Our institute started to use laparoscopic in-bag morcellation in 2016 and used both techniques until early 2017, when the contained laparoscopic morcellation technique was established as the new standard. Thus, the patients could not be randomised as this was a retrospective analysis from this phase of transition. In all cases, a presurgical gynaecological examination combined with transvaginal and abdominal ultrasound was performed. The presurgical cervical screening of all patients showed normal results. The indications for the procedure included bleeding disorders, adenomyosis, and uterine myomatosis. Written informed consent was obtained from all patients.

All surgical cases utilised a 12 mm optical trocar and a 12 mm auxiliary trocar inserted into the left lower abdomen. Depending on uterine size and mobility as well as additional adhesions, one (right lower abdomen) or two additional auxiliary 5 mm trocars (midline) were used. An additional uterine manipulator was only used in a few cases of very high uterine weight and immobility. Morcellation was achieved with the Rotocut G1 by KARL STORZ. In-bag morcellation was carried out with More-Cell-Safe by A.M.I. The surgical process in both approaches was identical until the dissection of the uterine corpus from the uterine cervix was completed. In LASH with uncontained morcellation, we then inserted the 12 mm morcellator blade through the incision in the left lower abdomen. After morcellation, meticulous sampling of the tissue fragments and a lavage of the surgical site was performed. In cases of bag use, the bag was introduced through the 12 mm trocar in the left lower abdomen to the abdominal cavity. The laparoscopic morcellation bag has a dual opening system, which allows two- port access with a protected optical trocar against spread cell dissemination. After opening the bag, the uterine tissue was placed inside and the bag fastened by pulling the drawstring. The closed opening of the bag was extracted through the same 12 mm incision. The second bag opening was then withdrawn through the incision in the umbilicus using a rendezvous technique with the 5 mm trocar in the right lower abdomen. Subsequently, the optical trocar was inserted through the umbilical opening into the bag which was then inflated the bag to 15 mm Hg. In the next step, the covered laparoscope was inserted through the umbilical port and the morcellator placed into the bag through the opening in the left lower abdomen under direct visualisation. Uterine tissue morcellation was performed as usual. After extraction of the morcellated uterine corpus, the morcellator and the optical trocar were removed, the umbilical opening of the bag closed, and the complete bag including disseminated cells, blood rests and tissue liquids extracted from the abdomen.Surgery then continued with short lavage and control of the surgical site. The detailed description of the use of the More-Cell-Safe by A.M.I. has been reported in the wider literature (Rimbach et al., 2015; Rimbach et al., 2016).

The statistical analysis was carried out with Datatab using an independent t-test and Levene test of variability. A PubMed database search using the keywords laparoscopic supracervical hysterectomy, electric power morcellation, in-bag- morcellation, cell dissemination, tissue retrieval was carried out in order to discuss the results. The patients in both groups were similar regarding indication, prior surgery, body mass index and average uterine weight.

## Results

### Surgical time

The fastest LASH with uncontained morcellation took 45 minutes, in a patient with a uterine weight of 55 grams. The fastest LASH with in-bag morcellation was performed also in 45 minutes, in a patient whose uterus weighed 56 grams. The longest duration in the group who received LASH with uncontained morcellation was 204 minutes for a uterus of 214 grams, and, in the group who received LASH with contained morcellation, the longest procedure was 250 minutes. for a uterine weight of 1370 grams .

**Figure 1:**
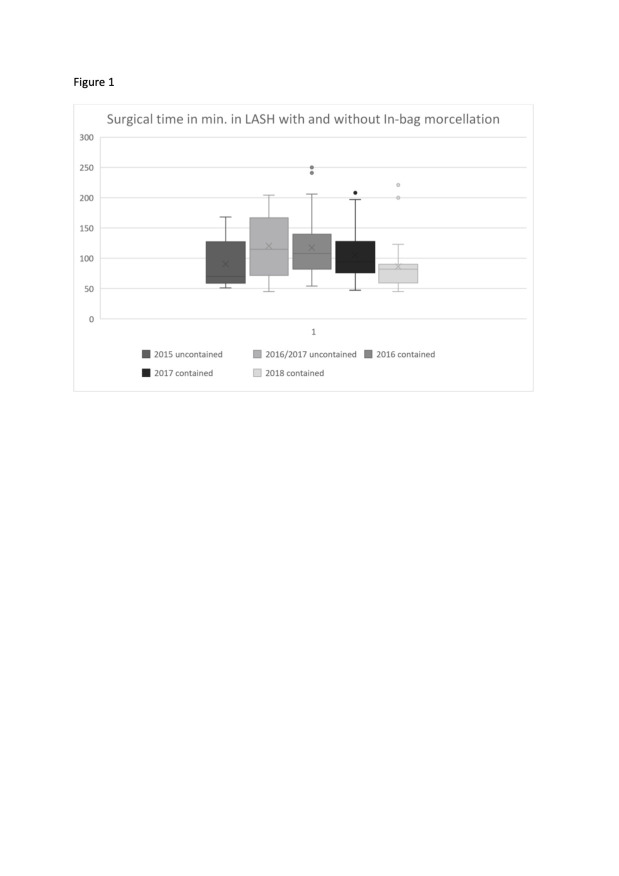
Surgical time in minutes for LASH with uncontained morcellation compared to LASH with in-bag morcellation.

The complexity differed in every surgery, as in some patients additional adhesiolysis, adnexal procedure or resection of peritoneal endometriosis was required. The mean surgical time for all cases was 103.5 minutes (45 – 250 minutes). The comparison of the mean surgical times of all surgeries between the group of LASH with in- bag morcellation (104.3 minutes) and the group of LASH with uncontained morcellation (101.4 minutes) was not statistically significant. The use of laparoscopic in-bag morcellation was not associated with overall additional surgical time. Analysing LASH with in-bag morcellation for the years 2016, 2017 and 2018 revealed a learning curve effect with the shortest mean surgical time over all included surgeries in the third year of bag use (86.2 minutes) and a significant decrease of surgical time compared to the surgical time in 2016 to 2017 (p 0.016) (Table I) (Figure 1).

**Table I:** Mean surgical times and uterine weights for LASH with and without in-bag morcellation. N = cases from 2015 – 2018.

n	Year / type of morcellation	Surgical time in minutes	Uterine weight in grams
30	2015 uncontained	90.5	166.5
17	2016 / 2017 uncontained	120.7	168.8
31	2016 contained	117.5	197.6
51	2017 contained	105.2	173.0
25	2018 contained	86.2	154.5
47	2015 – 2017 uncontained	101.4	167.7
107	2016 – 2018 contained	104.3	175.0

**Figure 2:**
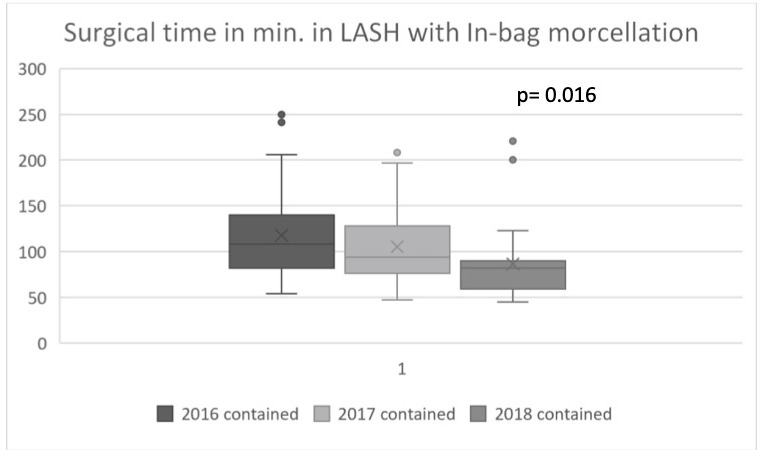
Improvement of surgical times in LASH with in-bag morcellation from 2016 - 2018.

In the first year of in-bag morcellation, the mean surgical time was higher than the mean time for all surgeries with uncontained morcellation. In the second year of bag use, the mean surgical time between both groups did not differ, and, in the third year of bag use, the mean surgical time with in- bag morcellation (86.2 minutes) was significantly shorter compared to the average surgical time of all LASH with uncontained morcellation (105.6 minutes). A positive learning curve effect with improvement in terms of duration of the surgeries from 2016 to 2018 (Figure 2) was observed.

**Figure 3:**
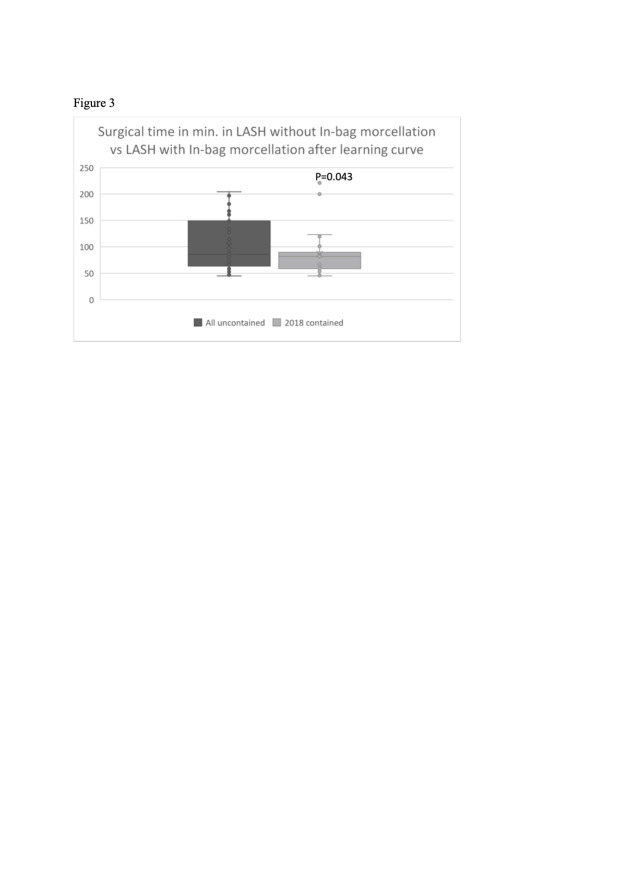
Surgical time in LASH with uncontained morcellation compared to LASH with in-bag morcellation after the learning curve process in minutes.

The results also show that the use of the laparoscopic system of in-bag morcellation in LASH decreases the overall surgical time when compared to the procedure with uncontained morcellation. Considering the learning curve, LASH with laparoscopic in-bag morcellation is faster than LASH with uncontained morcellation (Figure 3).

Laparoscopic supracervical hysterectomies in 2015 were carried out by the same surgeon. In 2016 and 2017, two additional surgeons started to perform the same procedure. This explains the difference in the mean surgical time in 2015 in comparison to 2016 and 2017 as the additional surgeons started at a different point of their individual learning curves. Furthermore, after the FDA warned against laparoscopic morcellation, the surgical time might have increased due to an even more meticulous surgical procedure.

### Uterine weight

Uterine weights ranged from a minimum of 13 g to a maximum of 1370 g. The mean weight was 172.77 g. The mean uterine weight in the LASH group with uncontained morcellation was 167.7 g, and in the group of in-bag morcellation 175.0 g. The uterus with a weight of 1370 g was successfully extracted from the abdominal cavity by use of laparoscopic in- bag morcellation without any complications. While the complete surgical time does not solely depend on the uterine weight, as many other factors like obesity, adhesions and other intraperitoneal comorbidities can have an effect on the surgical time, the longest duration of LASH in this study was reported in the case of the largest uterus.

### Blood loss

Blood loss was estimated by determining presurgical and postsurgical serum haemoglobin in g/dl in all patients. The postsurgical parameter was measured the first postsurgical day. The mean presurgical haemoglobin was 12.9 g/dl, while the mean postsurgical serum haemoglobin was 12.05 g/ dl. The mean blood loss was a difference of 0.85 g/dl between pre- and postsurgical serum samples. There was no significant difference between both groups (Table II).

**Table II:** Blood loss in LASH reported by mean pre- and postsurgical haemoglobin in g/dl.

n	Type of morcellation	pre	post	Diff
47	uncontained	12,8	12.0	0,8
107	contained	13.0	12.1	0,9

### Indications and histopathological results

In this study, symptomatic uterine myomatosis, symptomatic adenomyosis or a combination of both benign pathologies were indications for LASH. The patients reported bleeding disorders, dysmenorrhea, dyspareunia or pelvic pain. Transvaginal ultrasound revealed uterine fibroids and / or adenomyosis. Patients with additional retrocervical deep endometriosis were excluded. We also excluded all patients with suspicious cervical screening or suspicion of any malignant lesions.

Histopathological examination revealed adenomyosis in almost half of the patients (76/154; 49.38 %) and uterine myomatosis in 68.18 % (105/154) of the patients. In 36 patients, pathology reported a combination of adenomyosis and uterine myomatosis (23.38 %). Disseminated uterine leiomyomatosis was found in only 5 patients (3.25 %) and in 4 patients no pathology was detected (2.60 %). No occult malignant lesions were found in the extracted tissue (0/154; 0 %) (Table III). In 56/154 patients, adenomyosis was predicted by transvaginal 2D ultrasound examination. This is a rate of 73.68 %. In most of these patients a combination of uterine myomatosis and adenomyosis was found upon histopathological examination.

**Table III:** Histopathological findings after LASH.

Histological diagnosis*	LASH with no bag 2015	LASH with no bag 2016/17	LASH with bag 2016	LASH with bag 2017	LASH with bag 2018
No pathology	2	0	0	2	0
Uterine myomatosis	23	9	21	37	15
Adenomyosis	9	11	16	24	16
Adenomyosis und uterine myomatosis	4	3	8	14	7
Leiomyomatosis	0	0	2	2	1
Malignancy	0	0	0	0	0

### Complication rate including bag lesion rate

**Figure 4:**
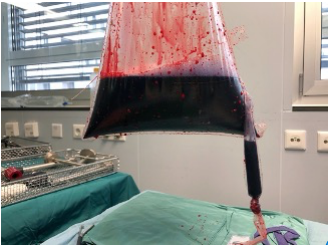
Intact bag after LASH with laparoscopic in-bag morcellation filled with blood, tissue liquid and blue dye.

All surgeries were completed without any major or minor surgical or direct postsurgical complications. In 3/107 (2.8 %) surgeries with in-bag morcellation a second bag was used as the first one was damaged during the laparoscopic placement of the bag. In these cases, the damage occurred before starting the morcellation process. There was no evidence of tissue or liquid spillage from the bag. No bags were damaged during morcellation, tissue or bag extraction after morcellation. The integrity of the bag after completed morcellation was verified in all patients by macroscopic examination of the bag filled with blood and tissue liquid. In some cases, the bag was filled with blue dye after the morcellation process in order to assess the bag integrity (Figure 4). No bag lesions were found after completed morcellation in any patients.

## Discussion

Intraperitoneal tissue morcellation during LASH can cause cell dissemination with subsequent benign or malignant peritoneal or retroperitoneal tumour growth. Depending on the original morcellated uterine tissue, parasitic peritoneal myomatosis, new onset peritoneal and retroperitoneal adenomyosis, endometriosis and endosalpingiosis, diffuse leiomyomatosis, the malignant transformation of initially benign tissue years after morcellation (Boes et al., 2011; Kiuchi et al., 2016; Krawczyk et al., 2016), and the dissemination of uterine sarcoma or carcinoma have been reported (Tulandi et al., 2016). The dissemination of occult malignant cells can worsen the prognosis of the disease (Raspagliesi et al., 2016). Cell, liquid and tissue spillage in uncontained LASH may result in subsequent intraabdominal tumour growth requiring secondary surgical interventions. These tumours can cause pelvic pain, vaginal bleeding, dysfunction of bladder, bowel and ureter, but can also remain asymptomatic for years.

In a review of 44 publications, the median time between surgery and diagnosis of parasitic myomas was 48 months (Van der Meulen et al., 2015). Various authors reported cases of new symptomatic iatrogenic peritoneal endometriosis 6 months after LASH (Sepilian and Della Badia, 2003; Brown, 2004; Decenzo, 2004). The overall risk of new peritoneal endometriosis after LASH with intraabdominal morcellation has been described to be 1.4 % by Schuster et al. (2012) However, in this comparative study, the authors only performed a second look laparoscopy in 12 symptomatic patients out of 464 patients included in the study. In another study with more than 1400 patients who underwent LASH with uncontained morcellation, a rate of 0.57 % of intraabdominal tumours was published (Donnez et al., 2007). Interestingly in this study all patients with symptomatic postsurgical lesions presented with adenomyosis in the initial histology of the uterine corpus. It would be of interest to compare these cases to the complete group of patients with initial adenomyosis. The true rate of iatrogenic peritoneal endometriosis and adenomyosis after uncontained morcellation of uterine tissue in LASH might be higher than the reported rates. The overall risk of unexpected malignant uterine tumours in LASH ranges from 0.13 % to 2.4 %. Various authors have reported retrospective studies with comparable results and a rate of mostly less than 1 % (Picerno et al., 2016; Tan et al., 2015; Theben et al., 2013). The discrepancies in these studies may be due to the different presurgical diagnostic approaches in order to exclude occult malignancies. As a presurgical standard procedure, a current cervical screening and transvaginal 2D ultrasound have been described. The combination with diagnostic hysteroscopy and endometrial biopsy might help to decrease the risk of occult malignancy. However, considering all presurgical and intrasurgical safety measures including the mentioned diagnostic tools, laparoscopic peritoneal washings and meticulous tissue sampling after morcellation, the risk of dissemination of benign and occult malignant cells will still exist for patients undergoing LASH with uncontained morcellation.

One possibility to avoid intraabdominal electric power morcellation is the extraction of the uterine corpus by posterior colpotomy or abdominal laparotomy. Both approaches are combined with additional incisions and might confer a risk of additional complications. Bogani et al. (2014) compared transvaginal tissue extraction with laparoscopic electric morcellation and found similar rates regarding surgical time, complications and blood loss between both groups. Boza et al. (2019) compared contained laparoscopic morcellation to transvaginal extraction after laparoscopic myomectomy. Both approaches are safe and feasible, while transvaginal tissue extraction was associated with shorter retrieval time and lower total costs. Ghezzi et al. (2018) described a technique of contained transvaginal tissue extraction after laparoscopic myomectomy in a total of 316 patients. The mean specimen weight was 154 g. They reported no intraoperative complications or bag lesions. However, 5.1 % of the patients had fever postoperatively. In a mini commentary on that publication, Miller discussed the limitations of the study by Ghezzi et al. He stated that 14.8 % of the patients underwent transabdominal tissue extraction due to virginal status, pouch obliteration or surgeon choice. A longer follow-up regarding postoperative complications like vaginal dehiscence or dyspareunia should be included in a multicenter randomised trial including patients who are obese and those with very large fibroids (Miller, 2018).

The use of laparoscopic containment systems in uterine morcellation represents a possibility to minimise the risk of cell dissemination while maintaining the benefits of laparoscopic surgery (Boruta and Shibley, 2016; Kanade, 2014; Clark and Cohen, 2018; Devassy et al., 2019). Although the use of in-bag morcellation systems is limited to a certain uterine size, laparoscopic in-bag morcellation in the supracervical hysterectomy of a uterus weighing greater than 1400 grams has been reported without bag lesions (Cohen et al., 2014; Krentel and De Wilde, 2017). The efficacy and safety of the contained morcellation varies depending on the respective material, technique and learning curve. Furthermore, the risk of tissue spillage and bag lesions depends on the surgical approach, the containment system, surgeon experience, and uterine size. Aoki et al. (2016) reported no bag lesions in laparoscopic in-bag morcellation, while Solima et al (2015) reported a rate of 33 % bag lesions after vaginal in-bag morcellation during total laparoscopic hysterectomy. Ikhena et al. (2016) reported no cytologic evidence of intraabdominal cell dissemination after enclosed morcellation in LASH. Contrarily, a rate of 9.2 % of liquid or tissue spillage after contained power morcellation was reported by Cohen et al in a multicenter prospective cohort study in 76 patients. Although containment bags were intact in all cases, leakage of applied blue dye was found (Cohen et al., 2016). Rimbach et al. (2015) reported negative peritoneal washings for muscle cells in all cases where bags were used in laparoscopic hysterectomy in a pig model, while positive cytology was found in 5 out of 8 cases in open power morcellation. Recently, Hong et al. (2020) reported a bag lesion rate of 13.3 % in contained manual morcellation during minimally invasive gynaecological surgery. Takeda et al. (2018) recently reported a dispersal of leiomyoma cells in most cases after laparoscopic myomectomy with contained tissue extraction and no bag lesion. This result might be traced back to cell dispersal in the moment of enucleation of fibroid as recently described by different authors (Asgari et al., 2020; Lambat Emery et al., 2019; Yu et al., 2018) and show that laparoscopic myomectomy and laparoscopic supracervical hysterectomy should be distinguished from one another when analysing this issue. The impact of small bag lesions during morcellation on the risk rate of intraabdominal cell dispersal should be evaluated in future studies. A final irrigation and suctioning procedure after uncontained, but also after contained morcellation, may reduce the risk of cell dispersal especially in laparoscopic myomectomy.

A supposed weak point of laparoscopic in-bag morcellation is the additional time required for bag placement and use. Rimbach et al. (2016) reported a time associated to the More-Cell-Safe use ranging from 8.5 to 26.5 min for specimens with a weight of 205 to 638 grams. Anapolski et al. (2016) described a mean time for bag insertion and preparation of 10.5 minutes, and a mean morcellation time of 10.5 minutes with an alternative bag system. The mean specimen weight ranged from 32-710 grams. Another technique for laparoscopic in-bag morcellation was described by Aoki et al (2016). The mean bag introduction time was 21.8 minutes, and the mean in-bag morcellation time was 11.5 minutes in 12 patients undergoing laparoscopic hysterectomy and myomectomy. Srouji et al. (2015) and Vargas et al. (2015) reported additional operative times ranging from 8.5 to 30 minutes. Winner et al. (2015) reported a 20-minute increase of surgical time in laparoscopic total hysterectomy with contained morcellation when compared to uncontained morcellation. The additional surgical time for contained morcellation depends on a variety of factors. General conditions such as the patient ´s weight and positioning, the positioning of auxiliary trocars, and the experience of the surgical team with complex laparoscopic procedures may all impact the duration of the surgical time. The main factors for the time associated with the bag use are the experience of the surgeon, the specimen size, and the bag system. In our retrospective analysis, we did not measure the time for bag placement and morcellation, but the complete surgical time from the first incision to the last suture. The results show that LASH with contained morcellation is as fast as LASH with uncontained morcellation.

When taking the surgeons ´ learning curve into account, the procedure with laparoscopic in-bag morcellation is even faster, as the time for bag use significantly decreases in relation to the experience with the system used. Our team started to use the More-Cell-Safe in 2016. The surgical time for LASH with in-bag morcellation decreased every year in relation to the growing experience of the three surgeons involved. Laparoscopic in-bag morcellation requires additional time for bag use, but on the other hand this technique saves surgical time as meticulous and time-consuming tissue sampling and extensive peritoneal washing after the morcellation procedure is not necessary. All dispersed cells and tissue fragments are contained within the bag and can be simply extracted at the end of morcellation. Laparoscopic in-bag morcellation enables to the use LASH as a safe and fast minimally invasive treatment option in patients with symptomatic uterine myomatosis and / or adenomyosis.

The groups are comparable as a standardised surgical procedure in our department was followed in all cases. A limitation of the study is the relatively small sample size of 47 interventions in the group of laparoscopic hysterectomy with uncontained morcellation. The variability of various surgical conditions might not be completely reflected. However, considering the disadvantages of uncontained morcellation it will be difficult to design a prospective study with larger sample sizes. In our department we established the use of contained laparoscopic morcellation as standard procedure in 2017. The exact times for the installation of the bag was not measured, as this has already been shown in previous publications and we compared the entire time of both surgical procedures. The results of this retrospective analysis show that laparoscopic supracervical hysterectomy with contained morcellation is a feasible technique, which can also be time-effective in the hands of the skilled gynaecological surgeon.

## Conclusion

LASH with laparoscopic in-bag morcellation is a safe and feasible minimal-access surgery even for very large uteri. The use of laparoscopic in- bag morcellation minimises the risk of inadvertent tissue dissemination during LASH. The overall surgical time does not increase in comparison with uncontained morcellation, yet actually decreases when the surgical learning curve is considered.
